# Supplementary material for: Repeated games with partner choice
Source: PLoS Comput Biol. 2025 Feb 4;21(2):e1012810. doi: 10.1371/journal.pcbi.1012810 (PMC11828350; doi:10.1371/journal.pcbi.1012810)
Supplement: S1 Text — The Supporting Information gives more detail regarding the theoretical model and the way it is simulated. It also gives theoretical results mentioned in the Main Text, with proofs. It is subdivided as follows. The Model. Calculating payoffs. Why these are the average payoffs, if we assume short-run equilibrium. With and without leaving in one setting. Frequencies in the matching pool and in the population as a whole. Histories and strategies. Finite state automata. Simulations. Different mutation procedures. Algorithm for the simulations. Theoretical results. No ESS. No strategy that is RAII. Pure strategies with a trust-building phase. (PDF) [file pcbi.1012810.s001.pdf]

Supporting Information for:

Repeated games with partner choice

Christopher Graser[1◕], Takako Fujiwara-Greve[2], Julián García[3], Matthijs van Veelen[4◕*]

1 Dana-Farber Cancer Institute, Harvard University, Boston, Massecheusetts, United States of America
2 Department of Economics, Keio University, Tokyo, Japan
3 Department of Data Science and AI, Monash University, Melbourne, Australia
4 Department of Economics and Business, University of Amsterdam, Amsterdam, the Netherlands

◕These authors contributed equally to this work.
* c.m.vanveelen@uva.nl

The model

We mostly follow the model setup in [1–3]. In these papers, the authors consider a repeated prisoner's dilemma with the option to leave (or, as they call it, with voluntary separation). In [1] the stage game payoffs are given by four parameters. We chose to restrict attention to prisoner's dilemmas with equal gains from switching, which means that we have only two parameters, if we also normalize the payoff of mutual defection to 0.

$$\begin{bmatrix} & C & D \\ C & b-c & -c \\ D & b & 0 \end{bmatrix}$$

Without loss of generality, we then chose $c = 1$, which is equivalent to dividing all entries by c. This implies that we interpret b as the benefit-to-cost ratio. This is the payoff matrix we use in the Main Text. Adding 1 to all entries does not change the equilibrium analysis, or the replicator dynamics, and because the simulations require non-negative payoffs, in order to be consistent with the payoff matrix used in the simulations, here in the Supporting Information we use the following equivalent payoff matrix.

$$\begin{bmatrix} & C & D \\ C & b & 0 \\ D & b+1 & 1 \end{bmatrix}$$

Assuming equal gains from switching limits attention to prisoner's dilemmas in which the cost of cooperation to oneself, and the benefits to the other, are independent of whether the other cooperates or defects. This is a real restriction. Normalizing the lowest payoff to 0, and setting the cost of cooperation to 1, on the other hand, is not a restriction of generality.

The model furthermore assumes that all pairs have an exogenous breakup probability of $1-\delta$, with $\delta \in (0, 1)$. Pairs can also be broken up because one of the players, or both,

choose to end the partnership. All individuals from broken-up pairs are re-matched before the subsequent stage game, so there are no costs associated with having to wait to play again. Re-matching happens uniformly at random among the set of all unmatched individuals between two stage games. We refer to this set as the *matching pool*.

The model in [1] is slightly different, in that the δ there represents the probability of an individual surviving between two stage games. If an individual dies, then the pair it was in is broken up. The dead individual is replaced by a new one, and both the newborn individual and the surviving individual from the pair go to the matching pool. This translates to an exogenous breakup probability of $1-\delta^2$. If the newborn individual simply inherits the strategy of the deceased individual, there is no difference between, on the one hand, surviving and, on the other hand, dying and being replaced. In that case, the model from [1–3] and the model in this paper are equivalent, up to the change from δ^2 to δ representing the probability for a pair not to be broken up exogenously. The latter allows for a bit shorter notation and representation of the results. Death and birth can also be reinterpreted as update events, at which individuals may switch to different strategies, based on the payoffs these strategies get in the population.

Introducing selection in [1–3] would imply that the offspring does not simply inherit the strategy of the deceased individual, or that only the current shares of the different strategies present in the population determine the probabilities with which they reproduce, but that also the payoffs of individuals in the current population determine the probabilities with which they pass on their strategy. In our model, introducing selection requires introducing replacement or update events altogether. We will introduce selection explicitly below, in the section that describes the simulations. All replacement events there will lead to the pair splitting up, but not all splitting up of pairs will be the result of replacement events. Before we do this, however, we describe how the payoffs are calculated for a given composition of the population.

Calculating payoffs

In repeated games without the option to leave, the expected payoff of a strategy can be computed by first computing the discounted payoffs for the strategy against every strategy present in the population, and then weighing those payoffs according to the shares of those different strategies in the population. In repeated games with the option to leave, this is no longer possible. Given that players can choose to leave, also in the absence of exogenous breakups, not all matches last equally long. That implies that who is matched to whom becomes partly endogenous, as some combinations of strategies stick together longer than others.

In order to be able to calculate the payoffs, we will have to assume that a strategy distribution is in a steady state, that is, it is in short-run equilibrium. Below, we will first give the formula for the average payoff of a strategy, and then we will show why the assumption that the population is in a steady state implies that the formula should have this form. In general, the repeated prisoner's dilemma has infinitely many pure strategies, with or without the option to leave. However, the populations we consider will always be finite mixtures of pure strategies. We will therefore refer to the strategies as s_i, for $i = 1, 2, ..., n$. This allows us to denote the frequencies of these strategies *in the matching pool* by a vector, x, where x_i is the frequency of strategy s_i in the matching pool.

For a combination of strategies s_i and s_j, T_{ij} will denote the number of periods that s_i and s_j play, if no exogenous breakup occurs. The expected duration of this match, given that the pair is broken up exogenously with probability $1-\delta$ after every round, is

$$L_{ij} = 1 + \delta + \cdots + \delta^{T_{ij}-1} = \sum_{t=0}^{T_{ij}-1} \delta^t = \frac{1-\delta^{T_{ij}}}{1-\delta}.$$

In order to compute the expected payoff V_{ij} of s_i against s_j, let a_t^{ij} denote the action profile at period t as strategies s_i and s_j meet, for $t = 1, ..., T_{ij}$. The payoff to strategy s_i, matched with strategy s_j, in round t is denoted by $u(a_t^{ij})$. Then,

$$V_{ij} = \sum_{t=0}^{T_{ij}-1} \delta^t u(a_t^{i,j}).$$

With x_i representing the stationary frequency of strategy s_i in the matching pool, the average per-period payoff of strategy s_i is given by

$$v_i(x) = \frac{\sum_{j=1}^n x_j V_{ij}}{\sum_{j=1}^n x_j L_{ij}}. \quad (1)$$

Why these are the average payoffs, if we assume short-run equilibrium

In short-run equilibrium, the shares of given pairs of strategies that find themselves in any given round should be constant. We denote the fraction of ordered pairs *in the population as a whole*, that consist of an s_i-player and an s_j-player, and that find themselves in the t'th round of their interaction, by $q_{ij,t}$. Because the game is symmetric, $q_{ij,t} = q_{ji,t}$ for any pair of strategies (s_i, s_j) and any t. It is also possible to derive the average payoff of an s_i-player using unordered pairs, but it will be more straightforward to think of ordered pairs.

In order for the frequencies $q_{ij,t}$ to be constant in the short-run dynamics, the share of (s_i, s_j)-pairs that find themselves in the t'th round of their interaction should be δ times the share of (s_i, s_j)-pairs that find themselves in the $t-1$'th round of their interaction – provided that $t \leq T_{ij}$. The reason is that between one period and the next, the probability that a group is not broken up exogenously is δ. All groups of (s_i, s_j)-pairs that find themselves in the $t-1$'th round of the game, and that are not broken up exogenously, become groups of (s_i, s_j)-pairs that find themselves in the t'th round – unless $t > T_{ij}$. For $q_{ij,t}$ to be constant in the short-run dynamics (see [4]), it therefore has to be equal to $\delta q_{ij,t-1}$, while $q_{ij,t} = 0$ if $t > T_{ij}$.

Because no strategy can end a partnership before the first round, the shares in the first round moreover must be proportional to the shares in the matching pool, if we assume short-run equilibrium. For ordered pairs, that means that $q_{ij,1}$ should be proportional to x_i times x_j.

If we then choose

$$q_{ij,t} = \frac{x_i x_j \delta^{t-1}}{\sum_{k=1}^n \sum_{l=1}^n \sum_{u=1}^{T_{kl}} x_k x_l \delta^{u-1}} \text{ for } 1 \leq t \leq T_{ij}$$

and

$$q_{ij,t} = 0 \text{ for } t > T_{ij},$$

then they are proper fractions, as they add up to 1, and all of these restrictions for them to be in short-run equilibrium are satisfied. These therefore are the shares of different types of (ordered) pairs that find themselves at different times in their interaction in short-run equilibrium.

Given that the population is in short-run equilibrium, the average payoff to a strategy in the population as a whole is constant over time. The average payoff to strategy s_i in the population for which x represents the frequencies of the strategies in the matching pool is given by

$$\begin{aligned} v_i(x) &= \frac{\sum_{j=1}^{n}\sum_{t=1}^{T_{ij}} q_{ij,t} u(a_t^{i,j})}{\sum_{j=1}^{n}\sum_{t=1}^{T_{ij}} q_{i,j,t}} = \\ &= \frac{\sum_{j=1}^{n}\sum_{t=1}^{T_{ij}} x_i x_j \delta^{t-1} u(a_t^{i,j})}{\sum_{j=1}^{n}\sum_{t=1}^{T_{ij}} x_i x_j \delta^{t-1}} = \\ &= \frac{x_i \sum_{j=1}^{n} x_j \sum_{t=0}^{T_{ij}-1} \delta^t u(a_t^{i,j})}{x_i \sum_{j=1}^{n} x_j \sum_{t=0}^{T_{ij}-1} \delta^t} = \\ &= \frac{\sum_{j=1}^{n} x_j V_{ij}}{\sum_{j=1}^{n} x_j L_{ij}}. \end{aligned}$$

This is formula (1) above.

With and without leaving in one setting

If we then were to revert back to a setting in which leaving is not allowed, then this would imply that we only allow for pairs of strategies s_i and s_j with $T_{ij} = \infty$, $L_{ij} = \frac{1}{1-\delta}$, and $V_{ij} = \sum_{t=0}^{\infty} \delta^t u(a_t^{i,j})$. Without leaving, and using the fact that x_i's are frequencies in the matching pool that add up to one, we find that the average per-period payoff becomes

$$\begin{aligned} v_i(x) &= \frac{\sum_{j=1}^{n} x_j V_{ij}}{\sum_{j=1}^{n} x_j L_{ij}} \\ &= (1-\delta)\frac{\sum_{j=1}^{n} x_j V_{ij}}{\sum_{j=1}^{n} x_j} \\ &= (1-\delta)\sum_{j=1}^{n} x_j V_{ij}. \end{aligned}$$

This is equal to the normalized, discounted payoffs of strategy s_i against the mix of strategies s_j, for $j = 1, ..., n$, where the discount factor is taken to be the probability δ with which pairs are not broken up exogenously. Calculating a stream of payoffs over time, and then normalizing therefore coincides with taking the average in the population – where normalizing means multiplying with $1 - \delta$. Therefore, in the setup that we created to accommodate for the possibility of leaving, if we choose not to allow for leaving, we are back in a situation that is equivalent to the standard setting without leaving. This also justifies comparing the cases with and without leaving within one and the same framework.

Frequencies in the matching pool and in the population as a whole

Let us go back to the repeated prisoner's dilemma with the option to leave. If we want to know how large the share of the population as a whole is that is playing strategy s_i, we can aggregate the frequencies of pair types over all strategies s_j that s_i could be matched with, and over all periods that a pair can be in. The shares of strategies s_i in

the population as a whole are given by a vector, x^*, where x_i^* is the frequency of strategy s_i in the population as a whole, for $i = 1, ..., n$.

$$\begin{aligned} x_i^* &= \sum_{j=1}^{n} \sum_{t=1}^{T_{ij}} q_{ij,t} \\ &= \sum_{j=1}^{n} \sum_{t=1}^{T_{ij}} \frac{x_i x_j \delta^{t-1}}{\sum_{k=1}^{n} \sum_{l=1}^{n} \sum_{u=1}^{T_{kl}} x_k x_l \delta^{u-1}} \\ &= \frac{x_i \sum_{j=1}^{n} x_j \sum_{t=1}^{T_{ij}} \delta^{t-1}}{\sum_{k=1}^{n} \sum_{l=1}^{n} x_k x_l \sum_{u=1}^{T_{kl}} \delta^{u-1}} \\ &= \frac{x_i \sum_{j=1}^{n} x_j L_{ij}}{\sum_{k=1}^{n} \sum_{l=1}^{n} x_k x_l L_{kl}} \end{aligned}$$

Frequencies in the matching pool therefore translate to frequencies in the population as a whole in a relatively straightforward way. Going in the other direction is not straightforward at all; for computing the frequencies in the matching pool from the frequencies in the population as a whole, there is generally no closed form expression; see [4]. The fact that the frequencies in the population as a whole and the frequencies in the matching pool can be different is part of what makes the game with the option to leave interesting, as it is an unavoidable symptom of the endogenous assortment that the setup with leaving allows for. This does however complicate the appropriate definition of the replicator dynamics, and it also makes the definitions of a Nash equilibrium and a Neutrally Stable State a bit more involved; see [4]. Theorem 1 in [4] moreover shows that a Neutrally Stable State, according to the definition in the same paper, is Lyapunov stable in the replicator dynamics. This is not the case for a Neutrally Stable Distribution as defined in [1].

Histories and strategies

Here, we give a formal definition of the game. This is not needed to understand the Main Text, or the main ideas here in the SI, but for the proofs of the theorems, a bit of additional formality can be useful.

We assume a set of players $I = \{1, 2\}$, an action space $A = \{C, D\}$, equal for both players, and a payoff function $u : A \times A \to \mathbb{R}$. A history at time t is a list of actions played up to and including time $t - 1$, where an empty pair of brackets is used to denote the null history. If $a_{t,i}$ is the action played by player i at time t, then these histories are:

$$\begin{aligned} h_1 &= () \\ h_t &= ((a_{1,1}, a_{1,2}), ..., (a_{t-1,1}, a_{t-1,2})), \qquad t = 2, 3, ... \end{aligned}$$

Sometimes we will also write $(h_t, (a_{t,1}, a_{t,2}))$ for a history h_{t+1}. We will also write $h_t^{\leftarrow}$ for history h_t, as seen from the perspective of player 2. The set of possible histories at time t is:

$$\begin{aligned} H_1 &= \{h_1\} \\ H_t &= \prod_{i=1}^{t-1} (A \times A) \qquad t = 2, 3, ... \end{aligned}$$

and the set of all possible histories is:

$$H = \bigcup_{t=1}^{\infty} H_t.$$

A pure strategy is a function $s : H \to A$ that maps histories to the action space.

Finite state automata

For the simulations, we will restrict attention to strategies that can be represented by finite state automata (FSAs). An FSA, or a Moore machine M, is a tuple $\{\{1, ..., n_M\}, \lambda_M, \mu_M\}$, where

- n_M is the number of states;
- $\lambda_M : \{1, ..., n_M\} \to \{C, D\}$ gives the output in every state when leaving is not possible;
- $\lambda_M : \{1, ..., n_M\} \to \{C, D, L\}$, with the restriction that $\lambda_M(1) \neq L$, gives the output in every state when leaving is possible; and
- $\mu_M : \{1, ..., n_M\} \times \{C, D\} \to \{1, ..., n_M\}$ gives the transitions as a function of the state, and the action of the other player.

Sometimes the formal definition of a machine also specifies in which state the machine starts, but because the states can always be renumbered so that the starting state is the first, we assume, without loss of generality, that the machine starts in state 1. Sometimes we will also order the remaining states, so that, if it plays against a copy of itself, then, in the absence of errors, it will transition from state i to state $i + 1$, until for the first time it goes back to a state it has already been in, or remains in the state it is currently in. Fig 1 in the Main Text gives a graphical representation of an FSA.

Simulations

Translating this framework into a finite population model that we can simulate to analyze long-run dynamics requires two further ingredients; we need to specify a selection procedure, and a mutation procedure.

To a large degree, we follow the approach taken in [5–7], and extend it to the setting with endogenous separation. As in those papers, strategies are represented by FSAs in our simulations. We do however depart from the model in those papers, in that we allow for the output in every state of the automaton – except for the starting state – to also take on the value *leave*, besides *cooperate* and *defect*. The starting state cannot have the output *leave*, as every strategy has to specify a stage game action for the first stage game after it is being matched.

Because the option to leave is not present in [5–7], they chose for all pairs in the population to start playing the repeated prisoner's dilemma at the same moment in time. In that setup, when all pairs have finished their repeated interactions (with independent, random durations), the entire population is updated based on the payoffs the individuals earned in the repeated game. This makes the selection dynamics a Wright-Fisher process. With leaving and re-matching, the beginnings of the repeated interactions are not synchronised, and therefore it will be hard to use the Wright-Fisher process. Here we use a Moran process instead, which implies that in each selection step, only one individual is replaced by the offspring of a random member of the population, where the probability for any individual to be the parent is proportional to their payoff. Selection steps in our simulations occur between stage games, and the relevant payoffs for reproduction are the payoffs from the last stage game. At a selection step, both partners in a randomly chosen pair are replaced. The pair is broken up, and the new individuals that replace them join the matching pool (see the description of the simulations in the section below for details). At a typical mutation step, only one member of a matched pair mutates, which in our simulations also happens between stage games, and independently of selection events. The pair is broken up, and both the mutant and its former partner join the matching pool. With extremely low probability, both partners in one pair mutate.

In this paper, the presence or absence of the option to leave is the only difference between the settings we compare. The simulation model without leaving in this paper is therefore different from the simulation model in [5–7], because here we do not have synchronised beginnings of the repeated games, and we have a Moran process instead of a Wright-Fisher process. The simulations in [8] also use a Moran process, but because they assume the low mutation limit, and because leaving is not an option there, they can calculate the fixation probabilities explicitly, rather than running agent-based simulations.

The exogenous break-ups that our selection and mutation processes bring about are, of course, relevant for the probability with which matched pairs are broken up in our simulations. To keep consistency with the basic theoretical framework, when talking about the simulations, δ will denote the probability with which the pair is not broken up exogenously. In the simulations, exogenous breakups happen if a pair is chosen for a selection step; if one or both individuals in a pair mutate; and if a pair is broken up exogenously without mutation or selection. The probabilities of the former two are kept constant across all simulation runs in Fig 5 in the Main Text. To vary δ, we only vary the rate at which exogenous break-ups occur that are unrelated to selection or mutation. The probabilities of mutation and selection events therefore put an upper bound on δ in the simulations, and in order to be able to go to relatively high δ's, we need to accept that replacement and mutation events are relatively rare.

Different mutation procedures

The mutation procedure we chose is a modified version of the mutation procedure in [5–7]. In their procedure, all mutations make single small changes; they can either change the output in a state, or delete or add a state, or change a transition. We chose a mutation procedure that also allows for multiple changes in one go. The reason to do this, is that with leaving, mutants that would have a selective advantage are sometimes multiple changes away from the resident automaton. In order not to have populations remain in disequilibrium states, because the mutation procedure cannot find the mutants that would have an advantage, we also allow for multiple changes in one mutation event.

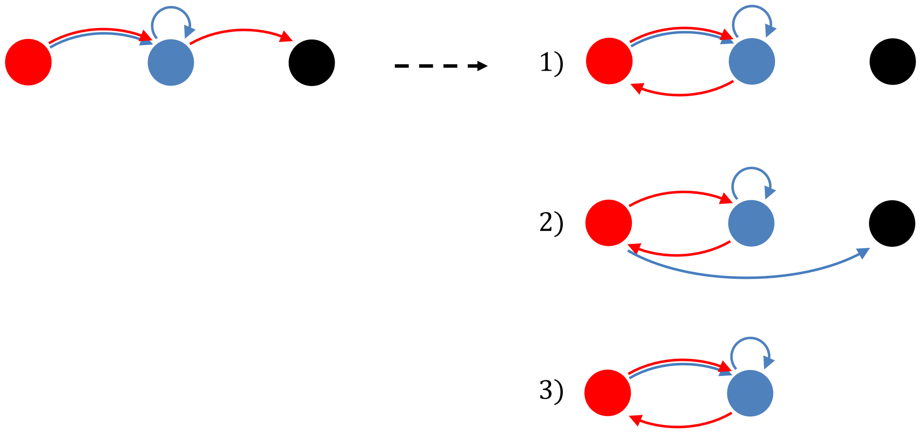

Fig 1. Three mutation procedures. A mutation may leave a state unreachable. In this example, a mutation to an outgoing arrow from state 2 leaves state 3 unreachable. With procedure 1) this unreachable state remains present, and future mutations may make it reachable again. With procedure 2) a random arrow is selected to point to the unreachable state. With procedure 3) the unreachable state is removed.

In the process of developing this mutation procedure we explored various different specifications. Since a mutation that redirects a link, or removes a state, can render one or more states unreachable, there are different options how to deal with those unreachable states. We tried out mutation procedures that after a mutation 1) keep the unreachable states around; 2) reconnect the unreachable states; and 3) remove all unreachable states (see Fig 1 here in the SI). With the first mutation procedure, even if states may not be reachable now, future mutations may reconnect to this "junk DNA". With the second mutation procedure, disconnected states are reconnected by choosing a single arrow from the component of the automaton that contains state 1. This may disconnect other states, so the procedure is repeated until no state is disconnected. In the simulations, the states in which the output is to leave still have outgoing arrows. That means that effectively, there can still be states that are unreachable. This mutation procedure does however on average produce far fewer (effectively) unreachable states.

We also tried different relative probabilities for adding or deleting states (see the code for details). All of these modifications affect the average size and complexity of the automata that evolve, and the share of unreached and neutrally-evolving states in the automata. We did however find the same qualitative results presented in this paper for all these versions of the mutation procedure (see Fig 2 here in the SI). The simulations reported in this paper all use the first mutation procedure.

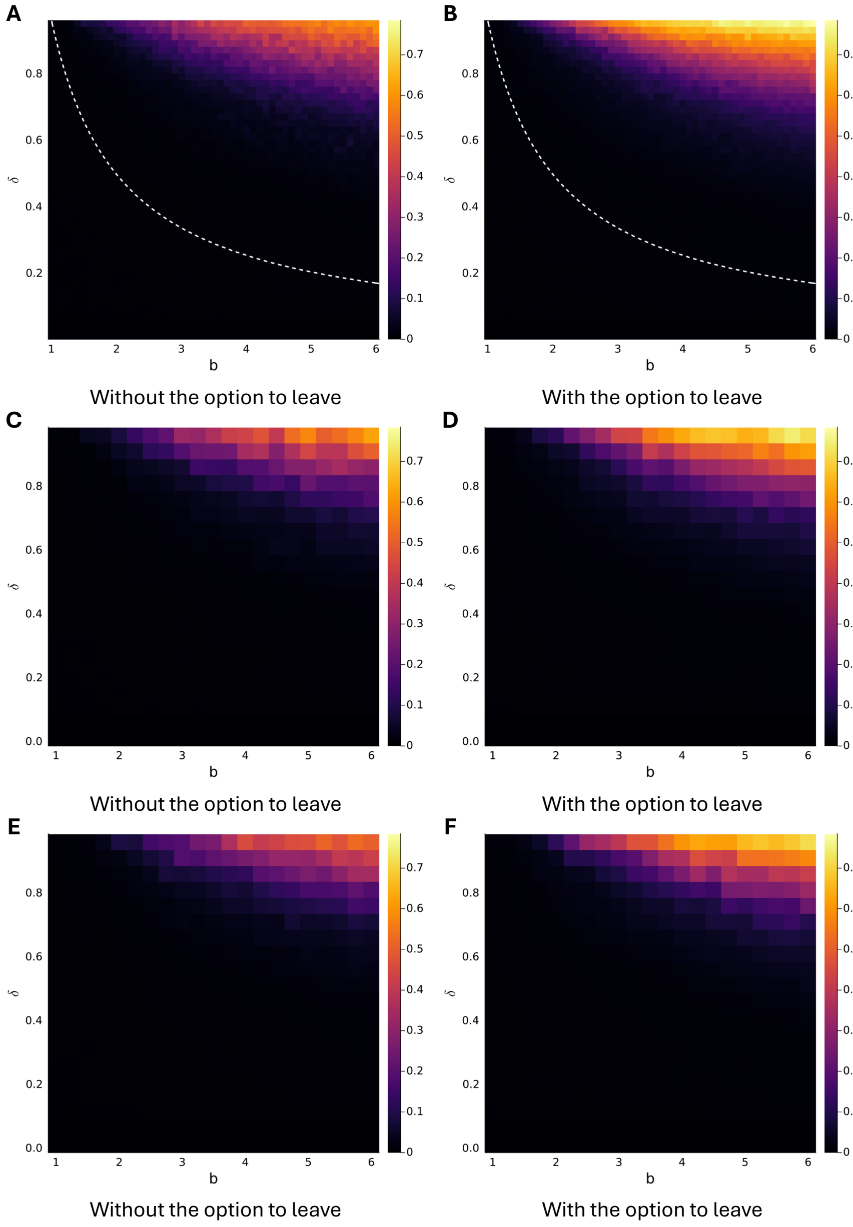

Fig 2. Simulation results with alternative mutation procedures. Panels A and D are the same as panels A and B from Fig 5 in the Main Text. For the simulations in panels B and E, disconnected states were re-connected by iteratively randomly choosing a single arrow from the component of the automaton that contains state 1, and pointing it to a randomly chosen disconnected state, until all states are reachable from state 1. For the simulations in panels C and F, disconnected states were simply removed. All parameters are as in Fig 5 in the Main Text. The ratio of average cooperation in panel E vs. panel B (calculated the same way as is done in the Main Text for panel D vs. panel A) is 1.3746. For panel F vs. panel C this ratio is 1.4519.

Algorithm for the simulations

A population state is characterized by a triplet of 1) a vector **a** of automata, 2) a vector **s** of the states that these automata are in, and 3) a matching **m** of the automata in **a**. Each period starts off with all automata in **a** being matched. If a match is dissolved, this match is removed from **m**.

In the simulations, there are different events that can bring about an exogenous breakup. For the algorithm, we denote the per-period probability that one individual in the population of $\frac{N}{2}$ pairs undergoes a mutation by α. Moreover, we denote the probability that a pair is broken up exogenously unrelated to a selection event or a mutation event by β, and lastly, we denote the per-period-per-individual rate of selection steps by γ. To reduce the amount of exogenous breakups brought about by selection steps, in each selection we replace both members of a matched pair at once. The probability that a pair is not broken up exogenously is, thus, given by $\delta = (1 - \frac{2\alpha}{N})(1 - \beta)(1 - \gamma)$.
The population state is updated by going through the following steps.

- Play
  - The actions {*cooperate*, *defect*} that the automata in **a** play against their current partner are determined by their current states **s**.
  - The automata update their states **s** according to their partners' actions.
  - Given its own action and its partner's action, each automaton in **a** is assigned a payoff.
- Selection
  - A draw from a binomial distribution with parameters $(\frac{N}{2}, \gamma)$ determines the number R of matched pairs whose members will be replaced. R matched pairs from the matching **m** are then selected uniformly at random and are removed from **m**.
  - A vector v of $2R$ new automata is generated by independently drawing from the automata in **a** with probabilities proportional to their payoffs.
  - The $2R$ elements in **a** that correspond to the members of the R matched pairs that were removed from **m** are then replaced by the elements in v. Note that this procedure allows for one automaton to produce more than one offspring in one period, and it allows for an automaton to produce offspring that replaces itself.
  - The corresponding $2R$ elements in **s** are set to 1.
- Endogenous break-ups
  - All matched pairs in **m** for which at least one member is in a state with a *leave* output are removed from **m**.
  - The states in **s** associated with the members of these removed pairs are set to 1.
- Exogenous break-ups
  - Independently, and with probability $(1 - \beta)$, matched pairs in **m** are removed from **m**.
  - The states in **s** associated with the members of these removed pairs are set to their initial state, 1.

- Mutation
  - One automaton in **a** is chosen uniformly at random.
  - With probability α, this automaton undergoes a mutation according to the procedure described in the previous subsection.
  - If the mutated automaton is a member of a matched pair in **m**, this pair is removed from **m** and the elements in **s** corresponding to the members of the removed pair are set to 1.
- Re-matching
  - Pairs of automata are drawn uniformly at random from the set of unmatched automata until all automata in **a** are matched.

Theoretical results

No ESS

There is no finite mix of strategies that is evolutionarily stable in the repeated prisoner's dilemma, with or without the option to leave. This is caused by the fact that in any population, there are histories that are never reached. This implies that it is always possible to construct a neutral mutant, that behaves identically to the resident after all histories that are reached, and differently in at least one history that is not; see also [9] and Proposition 6 in [6].

No strategy that is RAII

For any cooperative equilibrium, strategies that behave identically to the resident, but do not punish, can drift into the population as neutral mutants. Once such a neutral mutant has established itself as the new resident, it can subsequently be invaded by less cooperative strategies. Such indirect invasions are possible, whether punishment happens with defection or with leaving. For repeated games without the option to leave, this is observed in Theorem 7 of [6]. Here we give the equivalent result for the case with leaving.

Theorem 1. *No pure strategy that, when playing a copy of itself, ever plays C in the repeated prisoner's dilemma with endogenous separation is RAII.*

Proof. If a strategy cooperates against itself after some history h_t, it must disincentivise defecting at this history, either by leaving or by defecting in some future round. Otherwise, a mutant that defects at this history earns higher payoffs, and could invade directly. However, as the history in which a player defects after h_t is never reached, any strategy that does not punish this defection and otherwise behaves identically to the resident is a neutral mutant. Such a strategy could then be invaded directly by a strategy which behaves identically to the resident but defects at h_t. □

If the option to leave is not there, and a strategy does not always cooperate when it meets a copy of itself, then indirect invasions with an increase in cooperation are also feasible, provided that δ is sufficiently high. This is observed in Theorem 8 of [6]. The indirect invasion there is a neutral mutant that would cooperate (more), if the other initiates it, but does not initiate (additional) cooperation itself, followed by a mutation that initiates (additional) cooperation. For the second mutant to have an advantage, the δ needs to be high enough.

With leaving, here we present a simpler result, which is that full defection can always be invaded. It is simpler, in the sense that the starting point is a population in which all strategies defect with all other strategies, while the result for the case without leaving takes as a starting point all strategies that fall short of full cooperation. On the other hand, while without leaving, there is a restriction on the δ for this indirect invasion to exist, with leaving there is no such restriction, and an indirect invasion exists regardless of the value of δ.

Theorem 2. *No mixture of strategies, in which all strategies always defect with all strategies present in the population, is RAII.*

Proof. In a population in which every strategy present never cooperates with any other strategy present, all strategies are also defecting in the first period. The strategy that defects and leaves in the first period (which we will denote with d_0) therefore is a neutral mutant of any population with universal mutual defection. If by random drift d_0 takes over, and becomes the new resident, then a strategy that defects in the first round,

and then stays, and cooperates forever after, earns a payoff of 1 against d_0, as does d_0 against d_0, and as does d_0 against this strategy, while this strategy earns a payoff of $1 + \delta(b-1) > 1$ against itself. Any mix with universal defection therefore can be invaded indirectly. □

Theorems 1 and 2 combined imply that, also if leaving is allowed for, there is no pure strategy that is RAII. These are the equivalents of Theorems 7 and 8 in [6], which do the same for the case without leaving. The same paper also contains extensions of these two theorems to finite mixtures of strategies. A similar extension of Theorem 1 above can be made for the case with leaving. Theorem 2 already includes mixtures as it is, and does not need extending.

Given that no strategy exists that is RAII, the best we can do, is find strategies that are NSS. This is what we will look for below.

Pure strategies with a trust-building phase

In this section we translate a result from [1] to fit our simplified model with equal gains from switching, and an exogenous breakup rate rather than an individual death rate. Strategies c_T, that cooperate after a trust-building phase of length T, are defined in [1] as follows.

Definition 3. *For any $T \in \mathbb{N}_0$ let c_T be a strategy that*

- *plays D in period t if $t \leq T$,*
- *plays stay after round $t = 1, ..., T$ if and only if (D, D) is observed in round t,*
- *plays C in period t if $t \geq T + 1$,*
- *plays stay after round $t = T + 1, T + 2, ...$ if and only if (C, C) is observed in round t.*

Note that rounds of play in [1], and in this paper, are indexed starting at $t = 1$, so that strategy c_0 has no trust-building phase. Also, it is possible to give alternative, but equivalent definitions of a trust-building strategy c_T – where equivalent means that the alternative definition prescribes the same behaviour, not just when matched with c_T, but when matched with any possible strategy. Alternative definitions would specify actions differently only for histories that simply cannot occur, given that one plays according to c_T oneself.

In the simulations, strategies are encoded as FSAs that only respond to actions by the other player. This restricts their response to a history of length t ending in (D, D) to be the same as the response to the same history, but now ending in (C, D). Note, however, that, since output is deterministic, only one of them can occur, which makes responses to histories that cannot occur entirely inconsequential. Although the FSA representation in Fig 1 in the Main Text, for example, is not the exact same strategy as c_1, as defined in [1], it will play exactly the same as c_1, defined above, when matched with any possible strategy.

Because our stage game is a bit simpler than the stage game in [1], and because we have an exogenous breakup probability, rather than a probability for an individual to die, we can also give a simpler version of their Proposition 1, with a simpler proof. Our simpler version does however include an explicit formula for the thresholds over which c_T's become equilibria. These are depicted in Fig 3 here in the SI. Our version also includes the straightforward extension that, *below* the threshold, no equilibria exist that start cooperating before round $T + 1$.

Theorem 4. *(Fujiwara-Greve & Okuno-Fujiwara, 2009)*

1. *c_T is a Nash equilibrium if $b \geq \frac{1-\delta^{T+1}}{\delta-\delta^{T+1}}$.*

2. *All equilibria start with at least $T+1$ rounds of defection if $b < \frac{1-\delta^{T+1}}{\delta-\delta^{T+1}}$.*

Proof. Any other strategy s' that does not do the same against c_T as c_T does against c_T would have to deviate by either

1. playing leave if (D, D) is observed in some round $K \leq T$,

2. playing C in some round $K \leq T$

3. playing D in some round $K \geq T+1$, or

4. playing leave if (C, C) is observed in some round $K \geq T+1$.

Round K is the *first* round in which s' does something that is different from what c_T would do. All of these deviations can then be combined with additional, inconsequential changes beyond period K.

In order to be able to compare the payoffs of these alternative strategies, we first calculate the average per-period payoff of c_T in a population where everybody is playing c_T.

$$\begin{aligned} u\left(c_T, c_T\right) &= \frac{1 + \delta \cdot 1 + \cdots + \delta^{T-1} \cdot 1 + \delta^T \cdot b + \delta^{T+1} \cdot b + \cdots}{1 + \delta + \delta^2 + \cdots} \\ &= (1-\delta) \frac{\left(1-\delta^T\right) \cdot 1}{1-\delta} + (1-\delta) \frac{\delta^T \cdot b}{1-\delta} \\ &= 1 - \delta^T + \delta^T b = 1 + \delta^T (b-1) \end{aligned}$$

Then we go over the four ways to deviate described above

1. In a population in which everybody (else) plays c_T, the average per period payoff of a strategy s^1 that leaves in round $K \leq T$ is

$$u\left(s^1, c_T\right) = 1 < 1 + \delta^T (b-1) = u\left(c_T, c_T\right).$$

2. In a population in which everybody (else) plays c_T, the average per period payoff of a strategy s^2, that plays cooperate in round $K \leq T$, is

$$u\left(s^2, c_T\right) = 1 - (1-\delta) \frac{\delta^{K-1}}{1-\delta^K} < 1 < u\left(c_T, c_T\right).$$

This deviation therefore is worse than the deviation by s^1, which makes perfect sense, because playing C and being left is always worse than playing D and leaving.

3. In a population in which everybody (else) plays c_T, the average per period payoff of a strategy s^3 that plays D in round $K \geq T+1$ is

$$\begin{aligned} u\left(s^3, c_T\right) &= \frac{1-\delta^T}{1-\delta^K} + \frac{\delta^T - \delta^{K-1}}{1-\delta^K} b + \frac{\delta^{K-1} - \delta^K}{1-\delta^K} (b+1) \\ &= \frac{1-\delta^T}{1-\delta^K} + \frac{\delta^T - \delta^K}{1-\delta^K} b + \frac{\delta^{K-1} - \delta^K}{1-\delta^K}. \end{aligned}$$

For the comparison with $u\left(c_T, c_T\right)$, it may be even easier to rewrite this in a way that reflects that we can also consider the first T periods separately, and then consider the repeating pattern starting from period $T+1$:

$$\begin{aligned} u\left(s^3, c_T\right) &= 1-\delta^T + \frac{\delta^T - \delta^K}{1-\delta^K} b + \frac{\delta^{K-1} - \delta^K}{1-\delta^K} + \frac{\delta^K - \delta^{K+T}}{1-\delta^K} \\ &= 1-\delta^T + \frac{\delta^T - \delta^K}{1-\delta^K} b + \frac{\delta^{K-1} - \delta^{K+T}}{1-\delta^K}. \end{aligned}$$

Therefore, $u\left(c_T, c_T\right) \geq u\left(s^3, c_T\right)$ if and only if

$$\begin{aligned} 1-\delta^T+\delta^T b &\geq 1-\delta^T + \frac{\delta^T - \delta^K}{1-\delta^K} b + \frac{\delta^{K-1} - \delta^{K+T}}{1-\delta^K} \\ \delta^T b &\geq \frac{\delta^T - \delta^K}{1-\delta^K} b + \frac{\delta^{K-1} - \delta^{K+T}}{1-\delta^K} \\ \delta^T\left(1-\delta^K\right) b &\geq \left(\delta^T - \delta^K\right) b + \delta^{K-1} - \delta^{K+T} \\ \left(\delta^K - \delta^{K+T}\right) b &\geq \delta^{K-1} - \delta^{K+T} \\ \left(\delta - \delta^{T+1}\right) b &\geq 1 - \delta^{T+1} \\ b &\geq \frac{1-\delta^{T+1}}{\delta - \delta^{T+1}}. \end{aligned}$$

4. Because $u\left(s^3, c_T\right) > u\left(s^4, c_T\right)$ for obvious reasons (playing D and being left is always better than playing C and leaving), and because $u\left(s^2, c_T\right) < u\left(s^1, c_T\right) < u\left(c_T, c_T\right)$ for all combinations of δ and T, as seen above, this condition now guarantees that no other strategy can do better against c_T than c_T. This proves the first part of the theorem.

For the second part of the theorem, we define d_T as the strategy that plays defect for the first $T+1$ rounds, and leaves after round $T+1$. Let s be as a strategy that, when playing against a copy of itself, plays defect in the first T rounds, and plays cooperate for the first time in round $T+1$. The same calculations that gave us the threshold above imply that, if $b < \frac{1-\delta^{T+1}}{\delta-\delta^{T+1}}$, then $u\left(d_T, c_T\right) > u\left(c_T, c_T\right)$, and because $u\left(d_T, s\right) = u\left(d_T, c_T\right)$ and $u\left(c_T, c_T\right) \geq u(s, s)$, this implies that also $u\left(d_T, s\right) > u(s, s)$. Moreover, if $b < \frac{1-\delta^k}{\delta-\delta^k}$ for $k \geq 3$, then also $b < \frac{1-\delta^{k-1}}{\delta-\delta^{k-1}}$, so strategies with even shorter trust-building phases can also be invaded. □

Also Proposition 2 in [1] allows for a simpler version, given our simpler stage game. Besides translating it to the simpler setting, we also switch from the definition of neutral stability in [1] to the definition of neutral stability in [4]. For pure equilibria, the latter definition is more restrictive; the definition in [1] requires that no pure mutant can invade, while the definition in [4] also requires mixtures of strategies not being able to invade. Theorem 1 in [4] shows that being neutrally stable in the latter definition moreover implies Lyapunov stability in the replicator dynamics. Our Theorem 5 therefore is less general than Proposition 2 in [1], but it is also more specific about the bounds, and it shows that c_T satisfies a slightly more restrictive, and dynamically more relevant equilibrium concept.

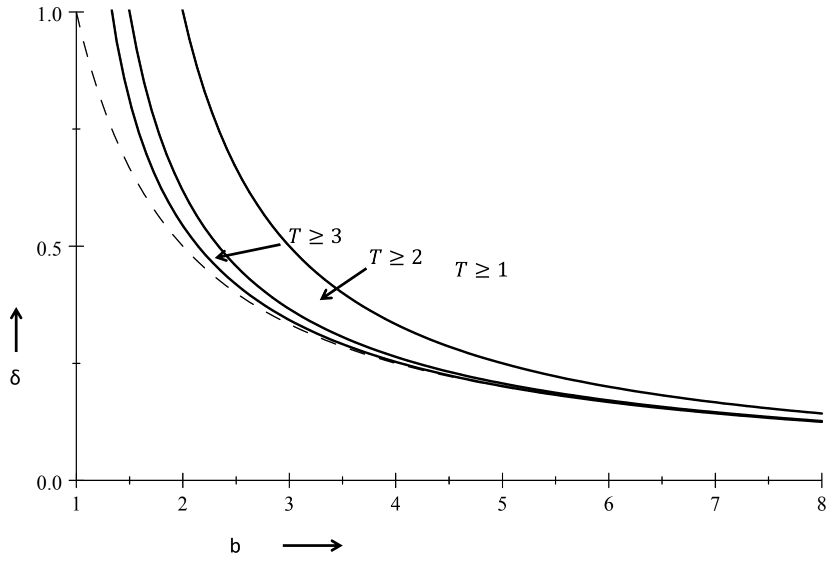

Fig 3. Pure c_T equilibria: NSS, not RAII. Right/up from the black lines is where different trust-building strategies c_T constitute equilibria. For any point right/up from the dotted line, given by $b = \frac{1}{\delta}$, there is some T such that c_T is a Nash equilibrium, as are all trust-building strategies with longer trust-building phases. Proposition 2 in [1], appropriately adapted, implies that these equilibria are also NSS.

Theorem 5. *(Fujiwara-Greve & Okuno-Fujiwara, 2009)*
c_T is an NSS if $b > \frac{1-\delta^{T+1}}{\delta-\delta^{T+1}}$

Proof. Here, we use Lemma 1 from [4]. This lemma states that if a strategy i never leaves when playing against itself, and $u(s_i, s_i) > u(s_j, s_i)$ for any strategy s_j such that a different history unfolds when strategy s_j plays with strategy s_i, then strategy s_i is a neutrally stable strategy. Using this lemma, we can then re-use the proof of Theorem 2, with strict inequalities for strategy s^3. □

Although c_T is an NSS when the condition in Theorem 5 is satisfied, it is, by Theorem 1, not RAII; there is always an indirect invasion with loss of cooperation.

Indirect invasions with an increase in cooperation are sometimes also feasible. A first mutant would play like c_T does, except that it would choose to stay whenever the other player cooperates, also in the first T rounds, and moreover that it would respond to the other player cooperating in any of those earlier rounds by also switching to playing C. The second mutant then could be c_K with $K < T - 1$, which implies it initiates cooperation at least 2 rounds before T. This would earn a payoff of $1 - \delta^K + \delta^{K+1}b$ against the first mutant, while $v(c_T, c_T) = 1 - \delta^T + \delta^T b$, which is also the payoff of the first mutant against itself. The second mutant would therefore have an advantage over the first if

$$b > \frac{1 - \delta^{T-K}}{\delta - \delta^{T-K}}.$$

The mutant with the largest advantage would be c_0, which starts cooperating immediately in round 1. Even though c_0 would itself not be a Nash equilibrium, it remains true that if any c_K can invade indirectly, then so can c_0. The condition for there to be a $K < T-1$ such that c_K can invade c_T indirectly, therefore, is that c_0 can invade indirectly. This condition is $b > \frac{1-\delta^T}{\delta-\delta^T}$, which is the same as the condition for c_{T-1} to be an equilibrium. In Fig 3 here in the SI, this implies that between the thresholds for c_T and c_{T-1}, c_T is an equilibrium, and while it can be invaded indirectly by a sequence that undermines cooperation, it cannot be invaded indirectly by a sequence that increases cooperation, without undermining it first. Between the threshold for c_{T-1} and c_T, one could therefore argue that c_T is more stable than the other trust-building strategies.

Punishing with leaving is always selected for when cooperation is selected for

Assume the following three strategies. Strategy 1 is g_n, which is Grim Trigger, preceded by a trust-building phase of n periods. In an FSA, this trust-building phase is represented by n states in which the output is defection, and in which the FSA always moves to the next state. After having gone through the n trust-building states, a cooperative state is reached, in which g_n stays as long as the opponent plays C, and a D state that is absorbing, and where g_n transitions to if the opponent played D (see Fig 2 in the Main Text for g_1). Strategy 2 is c_n, which is essentially the same strategy, but instead of punishing defections after the first n rounds with defecting forever, it leaves (see also the definition in the previous section). Strategy 3 is AllD. If x_1, x_2, and x_3 represent the shares of these three strategies in the matching pool, then their payoffs are:

$$
\begin{aligned}
v_1(x) &= \frac{\left(\frac{1-\delta^n}{1-\delta} + \frac{\delta^n b}{1-\delta}\right) x_1 + \left(\frac{1-\delta^n}{1-\delta} + \frac{\delta^n b}{1-\delta}\right) x_2 + \left(\frac{1-\delta^n}{1-\delta} + \frac{\delta^{n+1}}{1-\delta}\right) x_3}{\frac{1}{1-\delta}x_1 + \frac{1}{1-\delta}x_2 + \frac{1}{1-\delta}x_3} \\
&= 1 + \delta^n (b-1) - \delta^n (b-\delta) x_3 \\
v_2(x) &= \frac{\left(\frac{1-\delta^n}{1-\delta} + \frac{\delta^n b}{1-\delta}\right) x_1 + \left(\frac{1-\delta^n}{1-\delta} + \frac{\delta^n b}{1-\delta}\right) x_2 + \left(\frac{1-\delta^n}{1-\delta}\right) x_3}{\frac{1}{1-\delta}x_1 + \frac{1}{1-\delta}x_2 + \frac{1-\delta^{n+1}}{1-\delta}x_3} \\
&= \frac{1-\delta^n + \delta^n b (1-x_3)}{1-\delta^{n+1}x_3} \\
v_3(x) &= \frac{\left(\frac{1}{1-\delta} + \delta^n b\right) x_1 + \left(\frac{1-\delta^{n+1}}{1-\delta} + \delta^n b\right) x_2 + \left(\frac{1}{1-\delta}\right) x_3}{\frac{1}{1-\delta}x_1 + \frac{1-\delta^{n+1}}{1-\delta}x_2 + \frac{1}{1-\delta}x_3} \\
&= \frac{1 + \delta^n (1-\delta) b (x_1 + x_2) - \delta^{n+1} x_2}{1-\delta^{n+1}x_2}.
\end{aligned}
$$

Strategies 1 and 2 do equally well if $x_3 = \frac{b-1}{b-\delta}$; if we fill in $x_3 = \frac{b-1}{b-\delta}$ in the formulas for the payoffs above, we find that $v_1(x) = v_2(x) = 1$. From the formula for $v_3(x)$ above, we can immediately see that $v_3 \geq 1$, and that $v_3 > 1$ if $x_1 + x_2 > 0$, which holds because $x_3 = \frac{b-1}{b-\delta} < 1$, since $\delta < 1$. This implies that no trajectory of the replicator dynamics that starts at a population state x with $x_3 \geq \frac{b-1}{b-\delta}$ can ever reach a population state with $x_3 < \frac{b-1}{b-\delta}$. For that to happen, the trajectory would have to pass through some population state for which $x_3 = \frac{b-1}{b-\delta}$ at which the share of strategy 3 is decreasing. This would contradict the fact that the payoff of strategy 3 is higher than the payoff of the other strategies for all population states x for which $x_3 = \frac{b-1}{b-\delta}$.

The x here reflects shares in the matching pool. The first section of the SI details how these translate to shares in the population as a whole. Points on the simplices in the Main Text all depict shares of strategies in the population as a whole. The line where $x_3 = \frac{b-1}{b-\delta}$ here does have a constant share of strategy 3 in the matching pool, but that does not imply that the share of strategy 3 in the population as a whole is also constant. If the simplex in Fig 9 in the Main Text would have represented shares in the matching pool, the red line would have been parallel to the edge of the simplex at which $x_3 = 0$, but since the simplex represents shares in the population as a whole, it is not.

Code availability

The code used in our simulations is publicly available on Github:
`https://github.com/cjgraser/Repeated-Games-and-Partner-Choice`.

References

1. Fujiwara-Greve T, Okuno-Fujiwara M. Voluntarily Separable Repeated Prisoner's Dilemma. Review of Economic Studies. 2009;76(3):993–1021.

2. Fujiwara-Greve T, Okuno-Fujiwara M, Suzuki N. Efficiency may improve when defectors exist. Economic Theory. 2015;60(3):423–460.

3. Fujiwara-Greve T, Okuno-Fujiwara M. Diverse behavior patterns in a symmetric society with voluntary partnerships. Working paper. 2016; p. 1–46.

4. Izquierdo SS, Izquierdo LR, van Veelen M. Repeated games with endogenous separation. Working paper. 2021; p. 1–36.

5. van Veelen M, García J, Rand DG, Nowak MA. Direct reciprocity in structured populations. Proceedings of the National Academy of Sciences USA. 2012;109(25):9929–9934.

6. García J, van Veelen M. In and out of equilibrium I: Evolution of strategies in repeated games with discounting. Journal of Economic Theory. 2016;161:161–189.

7. García J, van Veelen M. No strategy can win in the repeated prisoner's dilemma: linking game theory and computer simulations. Frontiers in Robotics and AI. 2018;5:102.

8. van Veelen M, García J. In and out of equilibrium II: Evolution in repeated games with discounting and complexity costs. Games and Economic Behavior. 2019;115:113–130.

9. Selten R, Hammerstein P. Gaps in Harley's argument on evolutionarily stable learning rules and in the logic of "tit for tat". Behavioral and Brain Sciences. 1984;7(1):115–116.
