# Supplementary material for: Repeated games with partner choice
Source: PLoS Comput Biol. 2025 Feb 4;21(2):e1012810. doi: 10.1371/journal.pcbi.1012810 (PMC11828350; doi:10.1371/journal.pcbi.1012810)
Supplement: Attachment — Submitted filename: PLOS point by point R2.pdf [file pcbi.1012810.s002.pdf]

Our responses are in italics.

Reviewer #2: In "Repeated games and partner choice," Graser et al develop and analyze a model of repeated PD play with partner choice based only on the option to end one's current relationship. They compare this model to repeated PD play without the exit option and thus without endogenous partner choice. They do not consider partner choice without repeated PD play because their partner choice mechanism, which is quite compelling for reasons explained below, only works when repeated play is possible. They find that adding the exit option to repeated play supports the evolution of cooperation better than repeated interactions alone over a substantial region of parameter space.

This research is important for at least four fundamental reasons.

(1) It analyzes what happens when two mechanisms combine either to support the evolution of cooperation or not. We do have other examples of work combining mechanisms (e.g. Axelrod and Hamilton, 1981; van Veelen et al, 2012). But, it's also true that a bizarre acrimony has burdened work on the evolution of cooperation for a long time. This acrimony has little to do with science and almost everything to do with different tribes of researchers grinding their respective axes. This kind of thing happens. Why it happens in one field more than another? Who knows? Whatever the reason, the acrimony related to cooperation, I suspect, has meant that approaches combining mechanisms have received less attention than they would have in a counterfactual world without the acrimony. The current paper reminds us that, when it comes to the evolution of cooperation, hypothesized mechanisms are rarely incompatible. It also highlights how to move beyond the dysfunctional divisions that have marred the field in the past.

(2) Less generically, the current paper presents an especially convincing form of partner choice. It's straightforward and intuitive to argue that individuals probably do not pair off randomly, and this remains true even once we account for any biases in matching due to population structure. This raises the possibility that partner choice, however it works, creates some extra measure of assortment, and this is why it's interesting in relation to cooperation. That said, partner choice mechanisms can vary widely in terms of their informational assumptions. At one extreme, assume that everyone can directly and accurately observe the choices of everyone else. In such a world, everyone has a lot of reliable information about everyone. It's easy to imagine adding a partner choice mechanism that would allow cooperative individuals to identify each other and pair off endogenously. But, the amount of information in the system and the value of the information in the system are exogenously assured in a way that strains belief.

The current paper presents a partner choice mechanism at the opposite extreme. All individuals know is whether they like their current partners or not. Anyone who does not can end the current relationship and enter a pool of unmatched players available for random rematching. This pool is not representative of the entire population, and this bias is what creates the informational regularity that gives partner choice its force. In this case, the model does not strain belief at all. The informational requirements from the

perspective of the individual are minimal and completely reasonable. All I have to know is whether I like the guy I'm currently interacting with.

(3) With the use of FSAs to encode strategies for repeated play, the current model effectively allows an infinitely large strategy space. This is important because it increases confidence that any results are not artifacts of assumptions the researchers have made about the strategy space. The classic (and simplest) example of why assumptions about the strategy space matter, of course, is repeated PD with {ALLD,TFT} as a strategy space versus {ALLD,TFT,ALLC}. Other research (e.g. van Veelen et al, 2012) has suggested that groundless limits on the strategy space may be a general concern. But, the use of FSAs removes this worry, and this is another virtue of the paper.

(4) The paper opens with a very nice discussion of how the option to leave one's current partner has two countervailing effects on the evolution of cooperation. This raises the question, which effect is strongest and when? I found this framing to be quite compelling.

With these positives established, I would also like to offer several comments, questions, and suggestions. I begin with two suggested extensions for the paper. In my view, these extensions would provide a more complete picture of when and how repeated interactions and partner choice combine to support cooperation or not. I follow with a number of suggestions, comments, and questions about the writing. The paper varies a lot in terms of how clear and accessible different passages are. Some passages do a really great job of explaining ideas in an intuitive, precise, and accessible way. Other passages can be quite difficult to understand, and in some cases I'm simply not sure what the authors want to say. I flag the difficult passages so the authors can consider other ways of explaining whatever it is they want to explain. I offer specific suggestions when I have them.

It is much, much appreciated that the reviewer takes the effort not just to point to things that can be improved, but also to aspects that are already nice. And we totally concur with everything, including the description of the field! The experiences in the field also contribute to the fact that these reports feel like a breath of fresh air; high quality and non-partisan is unfortunately somewhat hard to come by.

Extensions:

(A) Include implementation errors in the simulations. Presumably, results remain similar for sufficiently low error rates, but where is the boundary? What happens as the amount of noise in the translation from strategies to behavior increases? I doubt the value of the paper can go down depending on the answers to these questions, but it can definitely go up. It's important to know the scope of the interaction between repeated interactions and partner choice, and varying the rate of implementation errors would almost certainly help the reader evaluate this scope.

The question of what the effect of errors is, is indeed super important! So much so, that Chris and Matthijs wrote a whole separate paper on that issue ([*https://tinbergen.nl/discussion-paper/6326/24-022-i-repeated-prisoner-s-dilemmas-with-errors-how-much-subgame-perfection-how-much-forgiveness-and-how-much-cooperation*](https://tinbergen.nl/discussion-paper/6326/24-022-i-repeated-prisoner-s-dilemmas-with-errors-how-much-subgame-perfection-how-much-forgiveness-and-how-much-cooperation)*). Without combining them, it is already quite a bit of work to track how errors affect the evolution of cooperation and how the option to leave affects the evolution of cooperation. Therefore we thought that it was better to first deal with those ingredients in separation. We do however totally share the intuition that for sufficiently low error rates, the results will not change very much. As a matter of fact, this "continuity in the error rate at an error rate of 0" is one of the results from the paper on errors that we linked to above. We now point to this paper, and two other papers on errors, at the end of the new Section 8.*

(B) In the supplement, the authors describe - briefly - the different mutational algorithms they used. The supplement should include more detail. I'm not suggesting additional analyses, just more information about the analyses the authors have apparently already done. First, the description of the different algorithms (e.g. bottom of p. 12) in the supplement is not sufficient; I really can't tell what the authors did from the all-too-brief descriptions. Second, the authors say that qualitative results remain the same regardless of the mutational algorithm used. It would be useful to include some of the other results in the supplement for readers to see this for themselves. Again, this will simply help readers evaluate the scope of how partner choice and repeated interactions combine.

We totally agree. This is now in SI A.9. We ended up describing the three different mutation procedures a bit more elaborately, and the SI now contains the equivalents of the main figures for the cooperation levels with and without leaving, but then for the alternative mutation procedures. These figures only have somewhat larger pixels.

The writing:

(i) On p. 3, the authors speak of reciprocity and cooperation evolving together. For my taste, this is a strange phrasing as it suggests that both reciprocity and cooperation are genetically encoded, under selection, and increasing in frequency. What actually happens is that reciprocal strategies are encoded and increasing in frequency, with cooperation as the behavioral result. Probably a matter of taste, but that was my reaction.

We totally share and apppreciate the concern to be precise in descriptions. We are open to suggestions for alternatives, but maybe we can also indicate how we landed on this compromise.

What we regularly see, is that reciprocity is mentioned as a ***mechanism*** *for the evolution of cooperation (a well-known example would be Nowak, 2006). We agree that this is a matter of taste, to some degree, but we are nonetheless somewhat uncomfortable with that, because the reciprocity itself is (also) the result of evolution. What allows it to evolve is repetition. So we wanted to step away from that way to describe what happens in some*

parts of the literature, and identify repetition as the key ingredient, or the cause. Given the point raised by the reviewer, we would guess that the reviewer will share our discomfort with labeling reciprocity as a mechanism for the evolution of cooperation.

Rather than identifying reciprocity as the cause and cooperation as the result, we therefore chose for a description in which they both evolve. Now, to the point raised by the reviewer, we do see that reciprocity and cooperation are not coevolving like, for instance, male and female traits within one species, and it is right to say that in the chain of causality, and in the chain of events in the simulations, cooperation follows reciprocity. However, the dichotomy between them is also not absolute. Not all reciprocal strategies are equally conducive to cooperation. For example, two (standard) tit-for-tat players will always cooperate with each other, but two suspicious-tit-for-tat players will never cooperate with each other, while both could be described as equally reciprocal. If we think of FSAs or strategies evolving, then reciprocity and for instance initial cooperation are still both part of the strategy.

Two further constraints are that 1) we do not want to spend a paragraph describing this in detail when we mention this in a classification or comparison, and 2) given that the broader context is still the debate on the evolution of cooperation, we also do not want to describe those models as models of the evolution of reciprocity – with cooperation as a side effect – because that could create the wrong impression that this is just an altogether different topic than for instance addressed by population structure models.

So all in all, we landed on this imperfect compromise. We are open to other compromises, but so far this maximizes how we would weigh the different ingredients.

(ii) On p. 5, it seems quite strange to call the initial string of defections the "trust-building" phase. The citations suggest that others have already established this terminology. I don't know. But, it's a strange label given we're talking about starting off a relationship with a bunch of defections. Consider a different label or a brief explanation of the logic behind the term "trust-building" in this context.

We agree, and yes, this is indeed a more or less established term. The idea is that it is the staying together (and not the defecting) that serves as an investment to induce cooperation afterwards. This is referred to as trust-building, but the reviewer is totally right to say that this is not what people would typically think of when using this term. We added a few lines acknowledging this, and confirming that we also use this label because it is used elsewhere too.

(iii) On p. 5, the authors say they "translate" a result from a 2009 paper in the Rev of Econ Studies. What does this mean?

That is indeed not well defined. We did also not locate exactly where in the SI the result that this refers to can be found. We now changed the wording, but are nonetheless happy to clarify what we had in mind. Because our stage game is a bit simpler than the stage

game in Fujiwara-Greve and Okuno-Fujiwara (2009), with fewer parameters, and because we have an exogenous breakup probability, rather than a probability for an individual to die, we were able to give a simpler version of their Proposition 1, with a simpler proof. Our simpler version moreover has an extra ingredient. While Fujiwara-Greve and Okuno-Fujiwara (2009) show that there always exists a threshold, our version includes a formula for the thresholds over which c_T's become equilibria. Our version also includes the straightforward extension that, below the threshold, no equilibria exist that start cooperating before round $T + 1$. The hope was that "translate" would capture all of those differences, but now we went for "restate a simpler version of". When the readers then go to the SI, they might be positively surprised to find that our restated version includes a few bonus extensions.

(iv) On p. 5, the authors provide a nice intuition about how the option to leave undermines the evolution of cooperation, but they decline to present an intuition about the upside until after they have described their model. I found this inconsistency a bit jarring. If they can provide an intuition about the downside before presenting the model, I guess they can provide an intuition about the upside.

This is also on point. We have adjusted this.

(v) The second half of p. 7 is confusing, and I didn't really understand the point the authors want to make.

We hope that the changes made, also in response to the other reviewer, address this, but if this part is still unclear, do let us know!

(vi) On the bottom of p. 8, the authors say that when a population is in equilibrium, punishment does not occur. This does not fit with the rest of the paragraph, which clearly suggests equilibria in which cooperation levels are positive, but maybe not as high as possible. Please clarify. Also, sentences of the following sort are often not very helpful: "The reason is similar to the reason without leaving." It's better to use more words, be less vague, and remove ambiguity.

Here we are a little unsure where the ambiguity is. What is true for sure is that we were imprecise in the Main Text regarding punishment in equilibrium. In pure equilibria, there is no punishment, but there are mixed equilibria in wich punishment is triggered; see for instance the c_0, d_0 equilibria from Fujiwara-Greve, Okuno-Fujiwara, & Suzuki (2015). We adjusted the Main Text accordingly (the SI that the Main Text refers to was already precise). If this is what sparked this comment, then we hope this is solved. What would not be true, is that the existence of a variety of equilibrium levels of cooperation implies that some of them must come with positive levels of punishment execution in equilibrium. If δ and b are high enough, then c_T is an equilibrium for all $T > 0$, and none of them have any punishment in equilibrium, while they all come with a different equilibrium level of cooperation.

The other reviewer suggested that a few figures with representative examples of indirect invasions would help, so maybe this also helps solve this problem. If not, we're happy to address this in a next round.

(vii) Similarly, the top of p. 9 talks about a neutral mutant attaining a "high enough" frequency, but this is less restrictive than the mention of "fixation" in the preceding paragraph. Please clarify.

Both are actually accurate; in those examples, the first mutant going to fixation is a sufficient condition for opening the door for a next mutant, but continuity implies that the door must already be open in an open interval before fixation happens; there is a threshold frequency such that, if the frequency of the first mutant exceeds that, the second mutant would attain a payoff that is higher than the average payoff in the population.

Just to be sure, we made the descriptions simple and consistent on and around what used to be page 9 (both pointing to the door being open after fixation), assuming that the discussion in Section 5 will add enough further detail to this.

(viii) I found most of p. 9 to be unclear and challenging.

This is shared with Reviewer #1, and hopefully the changes and the added figures help remedy this (but do let us know if not!)

(ix) I found the emphasis on b at the bottom of p. 11 to be a little strange. Looking at Fig. 1, variation in delta seems at least as important as variation in b, if not more important. Maybe the authors include this discussion simply because b, unlike delta, has no natural upper bound. I'm not sure. I just provide my reaction for the authors to consider.

This is a remark at a higher level of abstraction than our remarks. We did indeed think of δ having a natural upper and lower bound (1 and 0, respectively), while b does have a natural lower bound (below $b = c = 1$ there is nothing to be gained from cooperation), but no upper bound. When discussing the arbitrariness of the set of parameter combinations that we aggregate over, we therefore focused on the choice for the largest b we include. We could however also choose to leave out certain subsets of δ's, or combinations of δ and b, and that would also affect the aggregate. We do however hope that we can stay at this somewhat more mundane way of pointing out that some arbitrariness went into the 42%.

(x) Relative to Fig. 1, which provides more or less complete information, I didn't see a lot of added value for the exercise (pp. 11-12) in which the authors average over parameter space.

That is good to hear, but when we ourselves looked at panels (a) and (b) in what is now Fig. 5, from visual inspection, we would not have guessed that the aggregated amount of cooperation is 42% higher with leaving; we would have guessed that there is less of a difference. Our guess would moreover be that just by looking at the two panels, the

average reader would also underestimate the difference, and therefore we included this number. (An additional, non-scientific reason to like to keep this is that 42 is also the answer from a The Hitchhiker's Guide to the Galaxy).

(xi) On p. 12, the authors say that the combination of mechanisms lifts cooperation levels "across the board" relative to repeated interactions alone. Fig. 1c does not really support this claim because, for a subset of parameter space, the difference in cooperation levels is approximately zero. In any case, I don't see a need to say something like this. It's enough to show that cooperation levels increase for the complementary subset of parameter space. Moreover, it's interesting that the big effects occur when delta levels are high, a region of parameter space in which naive intuition might suggest that repeated interactions can support high levels of cooperation without any additional mechanism.

We agree that "across the board" can be thought of as including the region where there is virtually no cooperation, both with and without leaving. That is not what we meant to imply, so therefore we deleted the "across the board". If needed, we are happy to further adjust, but we hope that in combination with what one can infer by just looking at what now is Fig. 5c, everything is clear.

(xii) I found the middle of p. 12 confusing.

This was true for both reviewers, and we hope the new version is more clear.

(xiii) p. 14, "In the interior . . . population dynamics will be different too." This passage is quite vague.

Also here, we hope that with the changes, this is less vague.

(xiv) I found the analysis of escape times in the supplement very informative, and the authors might consider putting some of those results in the main text. I know space can be tight, but for me that section of the supplement was extremely useful.

We were happy to put it in the Main Text; it is the new Section 7. It is highlighted there, not because the text has changed, but because the location has.

(xv) The authors might consider a general, perhaps speculative discussion about when expanding the strategy space hinders versus helps the evolution of cooperation. After reading a paper like van Veelen et al (2012), one might think it only hinders. But this paper offers an example of it helping.

We agree that this is a useful perspective. We mention the expansion of the strategy space on page 15 and discuss the tradeoff; there are more mutants to consider, but also more residents that can be equilibria.

(xvi) The title could be more informative.

We agree that the title could indeed for instance include more info about what we find. We tried out a few titles and found that there is a tradeoff: more information without sacrificing precision makes the title much longer. We thus settled on a title that is at least short and unambiguous about what the topic is.
